# Clinical characteristics and proteome modifications in two Charcot-Marie-Tooth families with the *AARS1* Arg326Trp mutation

**DOI:** 10.1186/s12883-022-02828-6

**Published:** 2022-08-15

**Authors:** Helle Høyer, Øyvind L. Busk, Q. Ying. Esbensen, Oddveig Røsby, Hilde T. Hilmarsen, Michael B. Russell, Tuula A. Nyman, Geir J. Braathen, Hilde L. Nilsen

**Affiliations:** 1 Department of Medical Genetics, Telemark Hospital, PB 2900 Kjørbekk, 3710 Skien, Norway; 2 Department of Clinical Molecular Biology, University of Oslo and Akershus University Hospital, 1478 Lørenskog, Norway; 3 Department of Medical Genetics, Oslo University Hospital, 0424 Oslo, Norway; 4 Head and Neck Research Group, Division for Research and Innovation, Akershus University Hospital, 1478 Lørenskog, Norway; 5 Institute of Clinical Medicine, Campus Akershus University Hospital, University of Oslo, 1474 Norbyhagen, Norway; 6 Department of Immunology, Institute of Clinical Medicine, University of Oslo and Rikshospitalet, 0372 Oslo, Norway

**Keywords:** CMT2, Peripheral neuropathy, *AARS1*, Mitochondrial dysfunction

## Abstract

**Background:**

Aminoacyl tRNA-synthetases are ubiquitously-expressed enzymes that attach amino acids to their cognate tRNA molecules. Mutations in several genes encoding aminoacyl tRNA-synthetases, have been associated with peripheral neuropathy, i.e. *AARS1, GARS1, HARS1, YARS1* and *WARS1*. The pathogenic mechanism underlying *AARS1*-related neuropathy is not known.

**Methods:**

From 2012 onward, all probands presenting at Telemark Hospital (Skien, Norway) with peripheral neuropathy were screened for variants in *AARS1* using an “in-house” next-generation sequencing panel. DNA from patient’s family members was examined by Sanger sequencing. Blood from affected family members and healthy controls were used for quantification of *AARS1* mRNA and alanine. Proteomic analyses were conducted in peripheral blood mononuclear cells (PBMC) from four affected family members and five healthy controls.

**Results:**

Seventeen individuals in two Norwegian families affected by Charcot-Marie-Tooth disease (CMT) were characterized in this study. The heterozygous NM_001605.2:c.976C > T p.(Arg326Trp) *AARS1* mutation was identified in ten affected family members. All living carriers had a mild to severe length-dependent sensorimotor neuropathy. Three deceased obligate carriers aged 74–98 were reported to be unaffected, but were not examined in the clinic. Proteomic studies in PBMC from four affected individuals suggest an effect on the immune system mediated by components of a systemic response to chronic injury and inflammation. Furthermore, altered expression of proteins linked to mitochondrial function/dysfunction was observed. Proteomic data are available via ProteomeXchange using identifier PXD023842.

**Conclusion:**

This study describes clinical and neurophysiological features linked to the p.(Arg326Trp) variant of *AARS1* in CMT-affected members of two Norwegian families. Proteomic analyses based on of PBMC from four CMT-affected individuals suggest that involvement of inflammation and mitochondrial dysfunction might contribute to *AARS1* variant-associated peripheral neuropathy.

**Supplementary Information:**

The online version contains supplementary material available at 10.1186/s12883-022-02828-6.

## Background

Aminoacyl-tRNA synthetases (ARS) are essential enzymes that attach amino acids to their cognate tRNA molecules during the first step of protein synthesis [1]. There are 37 nuclear-encoded ARS for cytoplasmic and mitochondrial protein synthesis, each with a unique amino acid substrate specificity. The aminoacylation reaction occurs in two-steps; first the ARS enzyme forms a covalent bond with its amino acid substrate, and then, the amino acid is transferred to its cognate tRNA. The aminoacylated tRNA hybridizes with mRNA coding triplets, ensuring correct incorporation of amino acids in the growing polypeptide chain [1–4].

The cytosolic alanine-tRNA ligase (AlaRS), encoded by *AARS1*, attaches alanine to its cognate tRNA. Homodimerization of AlaRS is required for catalytic activity. Each AlaRS monomer consists of a class II catalytic domain, an editing domain and a C-terminal domain [3, 5].

Mutations in ARS genes have been linked to a wide spectrum of inherited human disorders. To date, recessive biallelic genotypes involving mutations in 34 of 37 ARS genes are associated with severe multisystemic or mitochondrial early onset diseases, while monoallelic heterozygous genotypes in ARS genes are associated with peripheral neuropathy [1–4]. Five of the ARS genes, *AARS1* (OMIM #613,287), *GARS1* (OMIM #600,794 and #601,472), *HARS1* (OMIM #616,625), *YARS1* (OMIM #608,323) and *WARS1* (OMIM #617,721), encoding alanyl-, glycyl-, histidyl-, tyrosyl- and tryptophanyl-tRNA synthetases, are strongly linked to peripheral neuropathy [6–10], while the evidence that *MARS1* (OMIM #616,280), encoding methionyl-tRNA synthetase, plays a role in peripheral neuropathy remains preliminary [11, 12].

Inherited peripheral neuropathy (HP: 0,009,830) is characterized by degeneration of motor and/or sensory nerves, muscle weakness and sensory disturbances in distal limbs, progressive motor loss, foot deformities and sometimes scoliosis. When both motor and sensory nerves are affected, the disease is referred to as hereditary motor and sensory neuropathy (HMSN) or more commonly Charcot-Marie-Tooth (CMT). CMT is further subdivided based on neurophysiological phenotype into CMT1 (demyelinating) and CMT2 (axonal), depending on whether the median motor nerve conduction velocity (MCV) is below or above 38 m/s [13, 14]. To date, more than 100 genes have been linked to peripheral neuropathies, of which the five *ARS* are the largest gene family [15]. Mutations in ARS genes predominantly cause adult onset axonal neuropathy, known as CMT2 or distal hereditary motor neuronopathy (dHMN), characterized exclusively by motor nerve pathology and symptoms [1, 3, 4, 16].

It is unclear why the ubiquitously expressed ARS enzymes cause tissue-specific late onset neuropathy [1]. In biallelic ARS disease, the null-mutation genotypes correspond well with the observed loss of ARS activity and impaired protein translation. In monoallelic ARS disease, in vitro aminoacetylation assays and yeast complementation assays have shown that most mutations impair enzyme function, but some increase catalytic activity. Furthermore, none of the neuropathy patients have null-mutations, pointing towards a different disease mechanism. Currently, two hypotheses have been proposed to explain these observations:1) mutant ARS alleles are dominant-negative inhibitors of ARS activity and function; or 2) mutant ARS alleles confer toxic gain-of function phenotypes mediated by non-canonical enzyme activity [1–4, 17].

The first report linking an *AARS1* variant to human disease was published in 2010. The report described two French families with dominant axonal CMT linked to the *AARS1* p.Arg329His mutation [6]. Since then, additional monoallelic mutations have been reported in families distributed around the world. To date, 17 mutations in *AARS1* have been linked to monoallelic disease [18]. Most of these mutations are in the catalytic domain, but some are in the editing and C-terminal domains [3, 18]. The p.Arg329His mutation, identified in Australian, British, Czech, French and U.S. families, is the most frequently reported [6, 19–23]. A second mutation, p.Arg326Trp, located three amino acids upstream the recurrent p.Arg329His mutation, was recently identified in a family with CMT2 [17]. Yeast mutants carrying the p.Arg329His and the p.Arg326Trp mutations do not grow in a genetic complementation assay [17, 19].

This paper describes two additional CMT2-affected families carrying the p.Arg326Trp missense mutation in *AARS1*. The results of high-resolution quantitative proteomics analyses of PBMC from four affected individuals and five healthy controls, suggest that inflammation and mitochondrial dysfunction might contribute to *AARS1* variant-related peripheral neuropathy.

## Methods

### Patients and families

The Norwegian families were referred to the genetic clinic at Telemark Hospital based on a diagnosis of peripheral neuropathy. Over a period of four years (2012–2016), the heterozygous *AARS1* variant NM_001605.2:c.976C > T p.(Arg326Trp) was identified in four individuals from three families. Family 2 and 3 were later identified to be branches of the same family and were merged to family 2.

The patients were examined in a genetic clinic (G.J.B or O.R). Additional family members were invited for a genetic consultation and DNA analysis. Nine affected individuals had a semi-structured clinical interview and a neurological re-examination. Cranial nerves, muscle weakness, reflexes, and sensation were scored according to the neuropathy impairment score (NIS) [24]. The controls were healthy adult blood donors or healthy adults from the genetic clinic, Telemark Hospital.

### Genetic analysis

Next-Generation Sequencing (NGS) was performed as a part of the routine clinical set-up. NGS included physical enrichment of an in-house panel containing neuromuscular genes according to Illumina TruSeq (2012–2015) or Illumina’s Nextera (2016–2019) standard protocols (Illumina Inc., San Diego, USA) and sequencing on Illumina HiScan SQ (2012–2015) or Illumina NextSeq 500 (2016-present) instrument according to standard procedures. The targeted bases consisted of all coding exons and flanking intronic, 5’ and 3’ sequences. The reads were mapped to the reference sequence (GRCh37/hg19) by BWA [25]. GATK (Genome Analysis Toolkit) was used for base quality score recalibration, indel realignment, duplicate removal and SNP and INDEL discovery [26–28]. Variants were annotated by Annovar [29]. Filtus software was used for bioinformatic filtering [30]. During the bioinformatic filtering 52, 91 or 99 peripheral neuropathies genes were included and analysed. The number of genes varied due to different time points of analysis (2012, 2014 and 2016). Gene lists are provided in Additional file 1. Identified variants were interpreted based on frequency data from the, GnomAD browser (https://gnomad.broadinstitute.org/) esp6500 (https://evs.gs.washington.edu/EVS/), and 1000 g (http://www.internationalgenome.org/), pathogenicity predictions through the Alamut interface (Interactive Biosoftware, Rouen, France) and reports in The Human Gene Mutation Database (HGMD) and the literature [18].

Verification of NGS results and co-segregation analysis in additional family members were performed by Sanger sequencing. Sanger sequencing was carried out using standard procedures and sequenced on the ABI3130XL (Life Technologies Ltd., Paisley, UK). CLC Main Workbench (CLC bio, Aarhus, Denmark) was used for sequence analysis.

### Haplotype analysis

Haplotype analysis was performed using the Applied Biosystems Linkage Mapping Set v2.5-MD10 (Life Technologies, Carlsbad, CA, USA). A 10 cM genome-wide scan including seven markers was conducted on the q-arm of chromosome 16. Electrophoretic length separation of the PCR products was performed on 3130XL Genetic Analyzer (Life Technologies, Carlsbad, CA, USA). Data were analysed by the program GeneMarker v. 1.85 (SoftGenetics LLC).

### RNA analysis

Blood was collected in PAXgene RNA collection tubes (PreAnalytiX GmbH, Switzerland) from four affected individuals and five control subjects. The samples were stored at − 80 °C until use. Total RNA was extracted using the PAXgene Blood RNA kit (PreAnalytiX GmbH, Switzerland) in accordance with the manufacturer’s recommendations. The RNA concentration was determined using an ND-1000 spectrophotometer (NanoDrop technologies, Saveen &Werner AB, Sweden); cDNA was synthesized using the High cDNA reverse transcription kit (Thermo Fisher Scientific, Waltham, USA) according to the provided protocols (Thermo Fisher Scientific, Waltham, USA). qRT-PCR was performed using QuantStudio 7 Flex Real-Time PCR Systems (Thermo Fisher Scientific, Waltham, USA). Pre-designed TaqMan® Gene Expression Assay (assay ID: Hs00609836_m1) was used for RT-qPCR and the 2 − ΔCT method of relative quantification was used to determine the fold change (FC) in gene expression analysis each performed in triplicates.

### Proteomics

Blood for protein analysis was collected on CPT-vacutainers from four affected individuals and five controls. Peripheral blood mononuclear cells (PBMCs) were isolated according to standard procedure for isolation and cryopreservation of PBMCs. For proteome analysis the cell pellets were thawed on ice and dissolved in 0.1% ProteaseMax™ Surfactant (Promega) in 50 mM NH_4_HCO_3_. The cells were lysed by vortexing, heating to 95^˚^C for 5 min followed by sonication for 30 min. The proteins were reduced, alkylated and digested with trypsin (Promega) according to ProteaseMax-protocol from the manufacturer. The resulting peptides were purified by C18-microcolumns and analysed by nanoLC-MS/MS using nEASY-LC coupled to QExactive Plus with 50 cm EASY Spray PepMap®RSLC-column and 120 min separation gradient. For protein identification and label-free quantification the LC–MS/MS data was searched with MaxQuant ver 1.6.1.0 against UniProt human (Oct 2017) database. Additional data processing and statistical analysis was done using Perseus ver 1.6.1.3. Further bioinformatic analysis and data visualization was done using softwares STRING (https://string-db.org) and Ingenuity Pathway AnalysisTM (Qiagen) using a 1.5-fold difference cut-off and q- and *p*-value limits 0.05.

### Measurement of alanine

Serum for biochemical analysis was collected on standard vacutainers for seven affected individuals and nine controls. L-Alanine levels in total plasma, which were deproteinized using 10 kDa Spin Column (ab93349, Abcam), was measured by using a commercially available kit (ab83394, Abcam) according to the manufacturer’s instructions.

## Results

### Patients and families

**Fig. 1:**
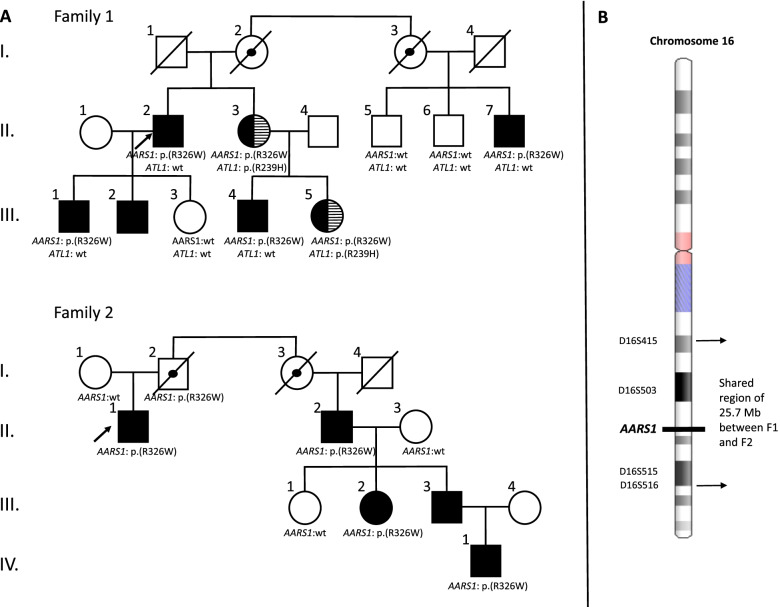
Pedigree and haplotype analysis. **A** Two CMT2 pedigrees and segregation of the *AARS1* and *ATL1* variants. Arrows = proband; black colour fillings = affected by CMT/CMT2; black lines filling = affected by HSP; black dot = obligate *AARS1* carrier, not clinically examined; wt = wild type. **B** Haplotype analysis results for six individuals from two families; Family 1: II-2 II-3 and II-7, Family 2: II-1, II.2 and III-2 depicted for chromosome 16. The *AARS1* gene and markers shared between the two families are marked to the figure. The ideogram of chromosome 16 was downloaded from https://www.ncbi.nlm.nih.gov/genome/tools/gdp according to the GRCh37/hg19 assembly

Figure 1A shows the pedigrees of the two CMT2-affected Norwegian families described in this study. Table 1 presents the clinical characteristics of six affected individuals from family 1 and a single affected individual from family 2. Table 2 presents the neurophysiological characteristics of affected individuals in family 1 and 2. The clinical and neurophysiological features of three deceased obligate carriers remain uncharacterized.

**Table 1:** Clinical characteristics of seven affected in the two CMT2 families

Family		Family 1						Family 2
**Individual**		**II-2**	**II-3**	**II-7**	**III-1**	**III-4**	**III-5**	**II-1**
Gender		M	F	M	M	M	F	M
Age at onset		18	7	45	35	36	32	22
Disease duration		44	64	22	15	8	8	32
Age at investigation		72	71	67	50	44	40	54
**Muscle wasting**^**1**^								
Underarm		0	0	0	0	0	0	0
Hand		2	0	0	0	0	0	2/1
Thigh		0	0	0	0	0	0	0
Leg		0	0	1	1	0	0	1
Feet		2	2	1	1	1	1	2
**Muscle weakness**^**NIS**^								
Elbow flexion		1	1	0	0	0	0	0
Wrist extension		1	0	0	0	0	0	0
Finger flexion		0	0	0	0	0	0	0
Finger spread		0	0	0	0	0	0	2/1^a^
Thumb abduction		3.25/3^a^	0	0/1^a^	0	0	0	3.25/1^a^
Knee flexion		0	0	0	0	0	0	0
Knee extension		0	0	0	0	0	0	0
Ankle dorsiflexors		3	2	3.5	1	1	0	3.25
Ankle plantar flexors		2	2	3.5	1	0	0	3.25
Toe extensors		3.75	2/3.25	3.5	3.5	1	0	3,5
Toe flexors		3.25	2	3.5	1	1	0	3.5
**Sensory loss**								
*Touch*^1^	Leg	1	0	0	0	0	0	0
	Feet	1	0	0	0	0	0	1
	Arm, hand	0	0	0	0	0	0	0
*Pain*^1^	Overarm	0	0	0	0	0	0	0
	Underarm	0	0	0	0	0	1	0
	Hand	0	0	0	0	0	1	1
	Thigh	0	0	0	0	0	0	0
	Leg	2	0	0	1	1	0	1
	Feet	2	0/1^a^	0	1	1	0	1/2
*Vibration*^1^	Hand	2	1	2	0	0	1	0
	2. finger	2	1	2	0	0	1	0
	Knee	2	2	2	0	1	1	0
	Ankle	2	2	2	0/1^a^	2	1	2
	1. metatarsal	2	2	2	2	2	1	2
	1.toe	2	2	2	2	2	1	2
*Proprioceptive*^1^	Toe	1	0	0	0	0	0	1
**Reflexes**^**1**^								
Biceps		2	2	1	0	0	0	0
Triceps		0	0	1	0	0	0	0
Brachioradialis		2	2	1	0	0	0	0
Patellar		2	2	1	0	2	0	0
Achilles		2	2	2	2	2	1	2
**Romberg**^**1**^		2	2	2	0	0	0	1
**Deformities**^**1**^								
Pes cavus		2	1	1	1	1	0	1
Hammertoes		0	1	0	1	0	0	0
Pes planus		0	0	0	0	0	1	0
Scoliosis		0	0	0	0	1	1	0
**NIS score**		**66,25**	**38.25**	**47**	**19**	**20**	**10**	**50.5**

-, not informative; ^1^0 = normal; 1 = mild/moderate affected; 2 = severely affected

*F* Female, *M* Male

^a^Asymmetrical

**Table 2:** Neurophysiology in affected carrying the *AARS1* variant

	Sex	Age (years) at		R/L	Motor nerves								Sensory nerves						EMG chronic denervation
		Onset	Exami-nation		Median		Ulnar		Peroneal		Tibial		Median		Ulnar		Sural		
					CMAP	CV	CMAP	CV	CMAP	CV	CMAP	CV	SNAP	CV	SNAP	CV	SNAP	CV	
Normal values →					4.0	49.0	4.0	49.0	3.0	41.0	3.0	41.0	12.0	46.0	17.0	47.0	17.0	44.0	
**Family 1**																			
II-2	M	18	57	R	**-**	**-**	-	**-**	**A**	**A**	**0.4**	**A**	**-**	**-**	**-**	**-**	**A**	**A**	**Present**
				L	**1.6**	**37.5**	4.9	50.2	**A**	**A**	**0.9**	**A**	**1.9**	50.0	**1.2**	**45.8**	**1.8**	**38.1**	**Present**
			59	L	**0.6**	**40.2**	5.6	**47.4**	**A**	**A**	**A**	**A**	**-**	**-**	**-**	**-**	**-**	**-**	**Present**
			61	L	**1.0**	**38.1**	5.8	51.1	**A**	**A**	**A**	**A**	**-**	**-**	**-**	**-**	**-**	**-**	**-**
			63	L	**1.0**	**35.8**	6.8	51.1	**A**	**A**	**A**	**A**	**-**	**-**	**-**	**-**	**-**	**-**	**Present**
			64	L	**0.4**	50.0	5.9	**44.1**	**A**	**A**	**A**	**A**	**-**	**-**	**-**	**-**	**-**	**-**	**Present**
			65	L	**0.6**	**43.8**	**3.7**	**45.3**	**-**	**-**	**-**	**-**	**-**	**-**	**-**	**-**	**-**	**-**	**Present**
			66	R	**0.2**	**46.2**	6.3	**41.7**	**-**	**-**	**-**	**-**	**-**	**-**	**-**	**-**	**-**	**-**	**-**
				L	**0.3**	**40.7**	6.7	**46.1**	**-**	**-**	**-**	**-**	**-**	**-**	**-**	**-**	**-**	**-**	**-**
II-3	F	7	58	R	-	**-**	-	**-**	**0.3**	**24.1**	**0.2**	**A**	**-**	**-**	**-**	**-**	**3**	**36.1**	**Present**
				L	-	**-**	-	**-**	**1.5**	**28.3**	**0.2**	**A**	-	**-**	**-**	**-**	**6**	**38.1**	**-**
			63	R	-	**-**	-	**-**	**A**	**A**	**0.2**	**29.6**	-	**-**	**-**	**-**	**3.3**	**46.2**	**Present**
				L	-	**-**	-	**-**	**0.1**	**37.1**	**0.3**	**39.8**	-	**-**	**-**	**-**	**2.9**	**50.0**	**Present**
			67	R	5.0	**41.7**	6.5	52.3	**A**	**A**	**0.2**	**A**	**2.7**	**-**	**1.0**	**-**	**2.6**	**43.1**	
				L	**0.8**	**39.0**	4.8	**45.8**	**A**	**A**	**0.6**	**A**	**2.5**	**-**	**1.7**	**-**	**2.1**	52.0	**Present**
II-7	M	45	60	R	7.8	**47.2**	-	**-**	**A**	**A**	5.4	**A**	17	**44.2**	**-**	**-**	**-**	**-**	**Present**
				L	**-**	**-**	-	**-**	**A**	**A**	**A**	**A**	**-**	**-**	**-**	**-**	**A**	**A**	**Present**
III-1	M	35	40	R	N	**↓**	N	**↓**	**A**	**A**	**A**	**A**	N	N	**-**	**-**	**↓**	**↓**	**Present**
				L	**-**	**-**	-	**-**	**A**	**A**	**A**	**A**	**-**	**-**	**-**	**-**	**↓**	**↓**	**-**
III-2	M	25	37	R	**N**	**40.0**	N	N	**↓**	**25.0**	**↓**	**31.0**	N	N	-	**-**	**3.8**	**37.0**	**-**
				L	**-**	**-**	-	**-**	**↓**	**29.0**	**↓**	**31.0**	**-**	**-**	**-**	**-**	**-**	**-**	**Present**
III-5	F	32	32	R	**3.8**	**45.5**	-	**-**	**1**	**34.6**	**2.5**	**34.2**	**8.5**	50	**5.6**	51.4	**3.8**	45.5	**Present**
				L	**-**	**-**	-	**-**	**0.4**	**33.7**	4.9	**32.6**					**2.4**	49.1	**-**
			42	R	**3.6**	**41.1**	4.8	**46.1**	**1.5**	**34.5**	3.7	**36.4**	**2.6**	46.8	**2.4**	58.1	**7.3**	51.7	**Present**
**Family 2**																			
II-1	M	22	38	R	**-**	**-**	-	**-**	**↓**	**-**	**-**	**-**	**-**	**-**	**-**	**-**	**↓**	**-**	**-**
				L	**-**	**-**	-	**-**	**-**	**-**	**-**	**-**	**-**	**-**	**-**	**-**	**↓**	**-**	**-**
			46	R	**↓**	**↓**	**↓**	**↓**	**-**	**-**	**-**	**-**	**A**	**A**	**↓**	**↓**	**-**	**-**	**-**
				L	**↓**	N	**↓**	**↓**	**-**	**-**	**-**	**-**	**↓**	**-**	**↓**	**↓**	**-**	**-**	**-**
II-2	M	-	67	R	**2.2**	**34.3**	9.2	49.5	**A**	**A**	**A**	**A**	**5.3**	**36.4**	**6.6**	**40.8**	**A**	**A**	**Present**
				L	-	-	-	-	**A**	**A**	**A**	**A**					**A**	**A**	**Present**
			69	R	**0.4**	**33.0**	-	-	-	-	-	-	**A**	**A**		–	**-**	**-**	**-**
				L	**1.3**	**31.0**	**3.4**	-	-	-	-	-	**A**	**A**	**1.9**	**41.6**	**-**	**-**	**-**
III-2	F	42	44	R	8.0	52.2	7.9	-	**2.5**	**33.3**	5.2	**35.5**	70.0	-	60.0	-	**6.2**	**40.4**	Normal^a^
				L	7.5	50.0	10.6	-	**0.9**	**31.6**	3.0	-	57.0	-	64.0	-	**6.1**	**50.0**	Normal^a^
IV-1	M	11	12	R	6.4	**44.2**	8.2	**-**	**1.7**	**35.4**	3.9	**39.2**	**2.4**	**-**	**3.9**	**-**	**2.8**	45.4	Normal
				L	**-**	-	**-**	**-**	**1.3**	**32.9**	**-**	**-**	**-**	**-**	**-**	**-**	**3.0**	45.9	Normal

Abbreviations: *Bold numbers/symbols* Abnormal values, *CMAP* Compound motor action potential (mV), *F* Female, *M* Male, *SNAP* Sensory nerve action potential (µV), *CV* Conduction velocity (m/s), *A* Absent evoked response,—= not measured, *R/L* Right/left, *N* Normal, exact value missing, ↓ Reduced value, exact value missing;

^a^EMG performed only in tibial anterior muscle

### Family 1

Clinical studies of the proband, II-2, demonstrated slowly progressing disease. At 18 years old, he was unsteady while cross-country skiing, especially downhill. At age 35, he was no longer able to walk on his heels, and at age 74 he used leg orthoses outdoors. II-2 was diagnosed with CMT2 at the age of 59.

The proband’s sister, II-3, had clinical symptoms including neuropathy and spasticity. As a child, she had poor balance, ran slowly and often tumbled, and later became a toe walker. At the age of 61, she experienced pollakisuria and urge incontinence with infrequent leakage. These gait and bladder problems progressed, and at the age of 71, she used crutches when walking, and a stair lift to climb stairs. Her clinical presentation is consistent with a diagnosis of both CMT2 and hereditary spastic paraplegia (HSP; OMIM #182,600).

II-7 demonstrated slowly progressive symptoms. At the age of 45, his gait was slightly unsteady and at the age of 50 he developed steppage gait. He was diagnosed with CMT2 at the age of 59.

III-1 had slowly progressive disease pathology. At the age of 35, he was unsteady walking on a steep uphill grade and had paresis for dorsiflexion of 1^st^ toes. Based on clinical presentation, he was diagnosed with CMT at the age of 50. Because neurophysiological data were incomplete, a differential diagnosis of CMT1 vs CMT2 was not made.

III-2 demonstrated slight unsteadiness and muscular cramps in legs and fingers at age 25. At age 30, he was unsteady, had problems walking on heels, and clinical examination revealed pes cavus and distal muscular atrophy in his legs. The neurophysiological data report when the patient was 37 years old is consistent with a diagnosis of CMT2.

At the age of 36 years, III-4 had a slight pes cavus, and lacked patellar and Achilles tendon reflexes. At the age of 44, he experienced leg cramps, reduced muscular strength and dysesthesia in the legs. While the clinical signs suggest a neuropathic phenotype, neurophysiological data were not available, so a precise CMT diagnosis was not possible.

III-5 had bilateral hip dysplasia and scoliosis. The hip dysplasia was treated surgically at age six. At age 40, she had spasticity in her legs and difficulty walking on her heels, and at the age of 41, she had urge incontinence. She was diagnosed with both HSP and CMT2.

### Family 2

At age 22, the proband II-1, had ankle weakness after soccer training, pes cavus and hammertoes. CMT was diagnosed at the age of 24. His clinical presentation at the age of 54 years is shown in Table 1. Neurophysiological examinations were conducted at ages 38 and 46, but did not provide exact measurements. Thus, a differential diagnosis of CMT1 vs CMT2 was not made.

The proband’s father, I-2, died at the age of 90 in a nursing home, after gradual health decline including memory loss. Neither the widow nor the proband could supply his medical history, however genetic analysis confirmed that he carried the *AARS1* variant.

The proband’s mother I-1 was examined at the age of 89. She denied neuropathic symptoms and the neurological examination was normal. She did not carry the *AARS1* variant.

The proband’s cousin, II-2, had a complex disease history, including surgery for lumbar disc herniation, right-sided drop foot, partial laminectomy, diabetes, coxarthrosis and surgery for bilateral hip implants. At the age of 69, he had bilateral neurological deficits in lower extremities, *i.e*. distal muscular atrophy, severe paresis, distally reduced sensation for pinprick with a border at ankles, reduced joint sense in 1^st^ toes and absent Achilles tendon reflexes. The late onset of clinical symptoms suggest CMT2, although this is not consistent with the median MCV for this patient, which was slightly lower than 38 m/s.

At age 42, the daughter of the proband’s cousin, III-2, had foot pain, dysesthesia in legs and ankle weakness. Neurological examination at age 44 revealed bilateral moderate paresis for ankle dorsiflexion, subtle paresis for knee and hip flexion, unsteadiness, absent Achilles tendon reflexes and pes cavus. The clinical and neurophysiological results are compatible with a diagnosis of CMT2.

At age 11, the grandson of the proband’s cousin, IV-1, had left ankle pain, especially when playing soccer, and had difficulty walking on heels. At age 13, he had pes cavus, paresis for dorsiflexion in ankles, absent Achilles tendon reflexes and distally reduced sensation for vibration, touch and pain with a border at ankles. The neurophysiological results are compatible with a diagnosis of CMT2.

### Genetic analysis

The heterozygous *AARS1* variant, NM_001605.2:c.976C > T p.(Arg326Trp) was initially observed in two affected individuals from family 1, the proband (II-2) and his sister (II-3). The *AARS1* variant segregated with the neuropathic phenotype (Fig. 1A).

The *AARS1* variant was identified in the proband (II-1) of family 2 and the daughter of the proband’s cousin (III-2) by NGS. In initial studies, III-2 was assigned as the proband of family 3, but genealogy studies revealed that families 2 and 3 are branches of the same family (described in the present report as family 2). The genotype was confirmed by Sanger sequencing. The DNA of 17 individuals in family 1 and 2 were analysed, of which 11 carried the *AARS1* variant. The *AARS1* variant segregated with the neuropathic phenotype in 10 individuals (Fig. 1A). In silico analysis using the Alamut interface showed that the Arg326 is a conserved residue in *AARS1*. The p.(Arg326Trp) substitution was predicted to have negative functional impact using SIFT [31], Align GVGD [32], Polyphen [33] and Revel [34] algorithm, and to be benign (no functional impact) by the Mutationtaster algorithm [35]. The variant was not present in our in-house database nor in the population databases GnomAD. The variant was situated in close proximity to the recurrent p.(Arg329His) mutation in *AARS1*.

In addition, II-3 and her daughter III-5 from family 1, who were affected by HSP, both carried the heterozygous variant in *ATL1* (NM_015915.4:c.716G > A p.(Arg239His). The *ATL1* variant was not present in other individuals from family 1 or in other patients in our in-house database. This variant has only been reported once in the population database GnomAD (1 of 251 004 alleles). Heterozygous pathogenic variants in the *ATL1* gene are linked to HSP or sensory neuropathy. The *ATL1* p.(Arg239His) variant has not previously been reported as pathogenic, but two other variants affecting the same codon p.(Arg239Cys) and p.(Arg239Leu) have been linked to HSP [36, 37]. Functional studies show that Arg239 is important for *ATL1* function [38–40].

### Haplotype analysis

Families 1 and 2 originated from the same geographical region in Norway and the *AARS1* variant had not previously been detected in our laboratory. In order to search for a common ancestral genetic variant, a haplotype analysis was performed for six individuals, including II-2, II-3 and II-7 from family 1 and II-1, II-2 and III-2 from family 2.

The results of the haplotype analysis showed that four markers (D16S415, D16S503, D16S515 and D16S516) distributed over 25.7 Mb on chromosome 16 are identical among the tested individuals (Fig. 1B). Although these data suggest that the two families trace back to a common ancestor, a link between families 1 and 2 could not be confirmed using available geneology data.

### AARS1 gene expression

**Fig. 2:**
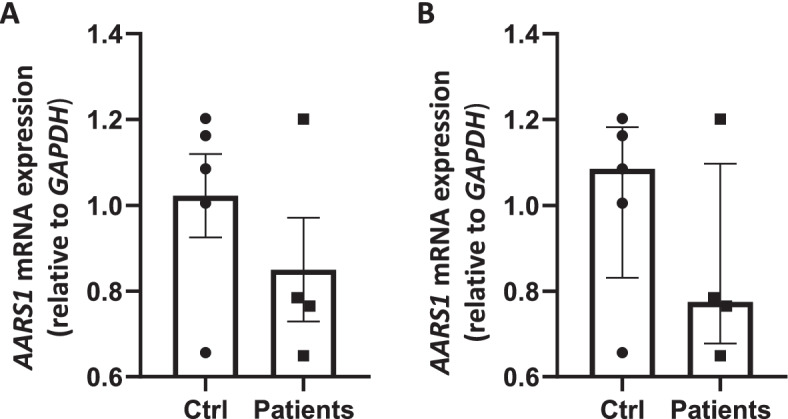
*AARS1* mRNA expression. *AARS1* gene expression level for four CMT/CMT2 patients and five controls (ctrl). Difference in gene expression is showed as fold change relative to *GAPDH* expression. **A** Mean ± standard error of the mean **B** Median with inter quartile range

As mutations sometimes affect stability of transcripts, we measured the expression of *AARS1* mRNA by RT-PCR. The analysis was performed using RNA samples from individuals II-2, II-3 and III-5 from family 1, individual II-1 from family 2 and five controls. A small quantitative difference between the affected individuals and the controls was detected (0.15 mean/0.25 median fold change) but this difference was not statistically significant (Fig. 2). With the exception of one outlier in each group, the samples clustered between 0.6 – 0.8 FC, and the controls clustered between 1 – 1.2 FC, with a difference of 0.4 FC. Thus, although a small statistically insignificant reduction in *AARS1* mRNA was detected in patients relative to controls, the results suggest, as expected, that transcript abundance and stability are not altered in patients carrying the *AARS1* p.(Arg326Trp) missense variant.

### Proteomics results

Because *AARS1* encodes an aminoacyl transferase, we postulated that a functional *AARS1* variant might affect translation of a range of proteins. Therefore, we performed a label-free quantitative proteome analysis on four affected individuals (II-2, II-3 and III-5 from family 1 and II-1 from family 2) and five healthy controls.

**Fig. 3:**
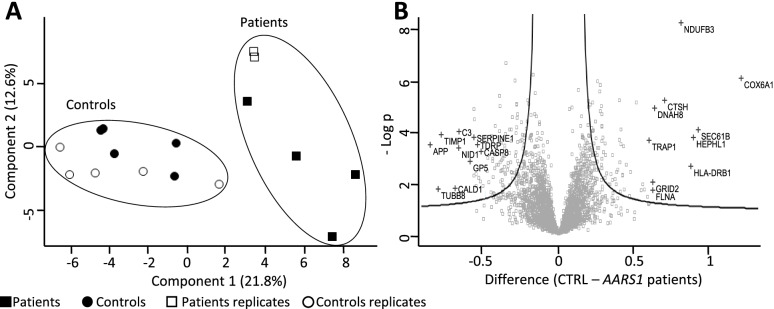
Proteome analysis. Label-free, quantitative proteome analysis of *AARS1* patient PBMC cells compared to healthy controls. **A** Principal component analysis shows clear separation of AlaRS patient proteome compared to healthy controls. **B** Volcano plot showing the 457 statistically significant differences in the proteomes (t-test, q < 0.05 and *p* < 0.05), ten most up- and downregulated proteins marked to the right and left, respectively

The analysis identified and quantified more than 4000 proteins. Principal component analysis of the data showed that the proteomes of the *AARS1* p.(Arg326Trp) patients cluster together and are clearly separated from the proteomes of healthy controls (Fig. 3A). Analysis of the proteome of four affected individuals compared to five healthy controls revealed a statistically significant difference for 457 proteins (t-test, q < 0.05 and *p* < 0.05) of which 247 were upregulated and 210 were downregulated (Fig. 3B and Additional file 2). The ten most strongly up and downregulated proteins are indicated in Fig. 3B.

Furthermore, the AlaRS protein was only slightly upregulated in patients relative to controls (Difference 0.12, *p* = 0.039, Additional file 2), showing that the *AARS1* mutation did not impair translation of *AARS1* mRNA. Also, the plasma concentration of alanine levels was unaffected (Additional file 3). These data do not support the idea that a build-up of alanine accumulates in cells carrying *AARS1* p.(Arg326Trp), due to a functionally deficient AlaRS protein.

**Fig. 4:**
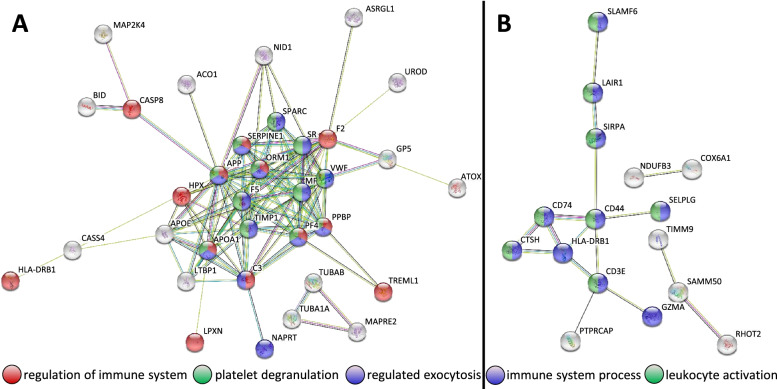
Protein–protein interaction network. Protein–protein interaction network visualized through STRING (https://string-db.org/). **A** Analysis with all proteins that are > twofold upregulated in proteomic data. **B** Analysis with all proteins that are > twofold downregulated in proteomic data

The most upregulated proteins in cells carrying *AARS1* p.(Arg326Trp) include amyloid beta (APP), metalloproteinase inhibitor 1 (TIMP1), complement C3 (C3), caspase-8 (CASP8) and copper transport protein ATOX1 (ATOX1), which are components of the systemic response to chronic injury and inflammation [41]. A striking number of mitochondria- associated proteins were among the top regulated proteins and interestingly, the most downregulated protein, COX6A1, situated in the mitochondrial complex IV (cytochrome c oxidase) is a CMT-associated protein (OMIM # 616,039) [42]. Protein–protein interaction network analysis showed that the upregulated proteins are involved in ‘regulation of immune system’, ‘platelet degranulation’ and ‘regulated exocytosis’ whereas downregulated proteins are involved in ‘immune system process’ and ‘leukocyte activation’ (Fig. 4).

**Fig. 5:**
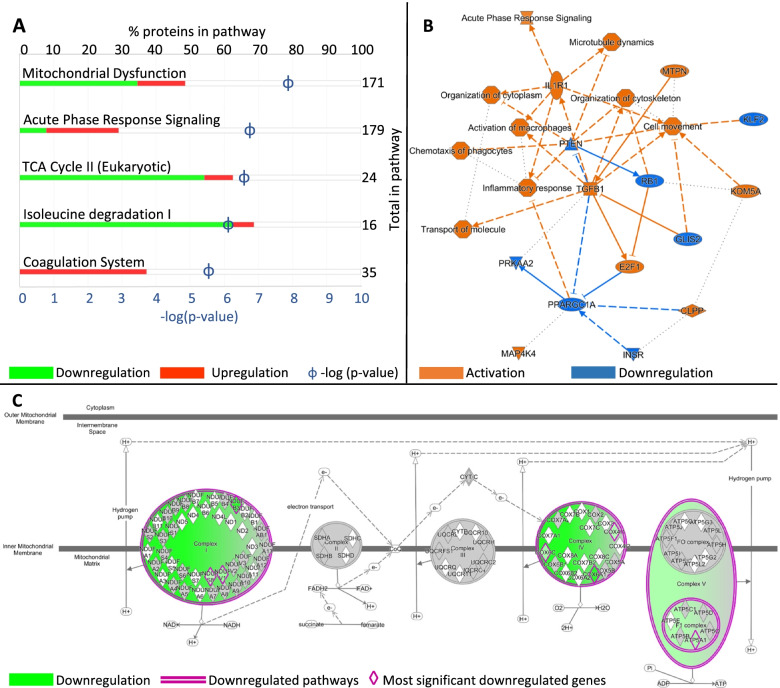
Ingenuity Pathway Analysis (IPA) analysis. IPA analysis of *AARS1* patient PBMC cell proteomes compared to healthy controls. **A** Canonical pathways. **B** Graphical summary of canonical pathways, upstream regulators and biological functions. **C** Illustration of oxidative phosphorylation

The Ingenuity Pathway Analysis (IPA) showed that 398 canonical pathways were significantly affected (Additional file 4) in cells carrying *AARS1* p.(Arg326Trp). IPA highlighted coordinated mitochondrial dysfunction, upregulation of the acute phase response signalling, downregulation of the tricarboxylic acid (TCA) cycle, downregulation of isoleucine degradation and upregulation of the coagulation system (Fig. 5A). Mitochondrial dysfunction was strongly highlighted in the IPA analysis, -log (*p*-value) of 7.9, involving both the most upregulated protein (APP) and the most downregulated protein (COX6A1). The IPA graphical summary showed activation of several canonical pathways and biological functions such as inflammatory response, macrophages, organization of cytoskeleton and cytoplasm and microtubule dynamics (Fig. 5B). Interestingly, this included an activation of the transcriptional factor E2F1, which is hyper-activated in a *Drosophila* model of CMT for *YARS* mutants [43]. Furthermore, we saw a striking downregulation of mitochondrial complexes I, IV and V, with no observed change in expression of complexes II and III (Fig. 5C).

## Discussion

This study presents clinical and molecular analyses of two Norwegian families with *AARS1* neuropathy. DNA sequence analysis confirmed that ten affected family members carry the *AARS1* variant p.(Arg326Trp).

### Clinical, neurophysiologic and genetic considerations

The phenotype and neurophysiological data were consistent with a diagnosis of CMT2 in seven individuals. In three individuals, it was not possible to assign a CMT subtype, because the neurophysiology data were incomplete or missing. For II-2 in family 2, MCV was below 38 m/s, suggesting demyelination and possible selective degeneration of fast-conducting motor nerve fibres. Although *AARS1* neuropathy is usually associated with CMT2, some patients carrying p.(Glu337Lys) also exhibit signs of demyelination [17]. The NIS of CMT-affected individuals increased slowly with age. Mildly-affected persons with CMT usually have a NIS score of 10–15, and markedly-affected individuals have a NIS score of 30–40 or higher [44].

The two pedigrees included four obligate deceased carriers, of whom individual I-2 from family 2 carried the *AARS1* variant. They were all reported by their family members to be unaffected. This might be due to reduced penetrance, mild phenotype and/or reporting bias. However, one cannot exclude the possibility that other genetic variants modified the phenotype of these deceased individuals. In the report by Weterman et al., one individual was clinically-unaffected at age 50, even though this individual’s neurophysiological data are consistent with a diagnosis of CMT2 [17]. Although, *AARS1* variants show variable severity and variable age of onset, we are not aware of prior reports of reduced penetrance variants in *AARS1* [6, 19, 22, 45, 46].

The *AARS1* p.(Arg326Trp) variant was previously linked to CMT in a Dutch family [17], while a Danish group described it as a variant of unknown significance (VUS) [47]. Haplotype analysis showed that families 1 and 2 are relatively closely related. Since this specific *AARS1* variant has only been identified in Northern Europe, it would be interesting to know whether Norwegian, Dutch and Danish CMT-positive families mentioned above share this same haplotype [17, 47]. During the last nine years, about 1,000 individuals with suspected peripheral neuropathy have been tested for mutations in *AARS1* at Telemark Hospital. The p.(Arg326Trp) variant of *AARS1* was not detected in other patients, indicating that this is a relatively rare variant. However, this variant might be underdiagnosed because of the variable phenotype. In clinical populations, CMT2 is likely to be underreported [48].

### Proteomic considerations

Because *AARS1* encodes an aminoacyl transferase, we postulated that *AARS1* variants might cause imbalanced translation, which could have an impact on translation of a range of proteins. To explore this possibility, we performed high-resolution, label-free quantitative proteomic analysis on extracts from PBMCs from four affected individuals and five healthy controls. Although sample numbers were low and two of the affected individuals also had an *ATL1* variant likely associated with HSP, the principal component analysis showed that *AARS1* patients and controls clustered separately, and the two HSP patients clustered together with two additional *AARS1*-positive patients. This indicates that the *ATL1* variant did not appreciably influence protein expression in PBMCs, although, the possibility of such an influence cannot be ruled out taking into account the low sample number.

The proteome analysis suggests prominent mitochondrial dysfunction in patients carrying the *AARS1* variant. Interestingly, mitochondrial dysfunction is not an uncommon event in axonal neuropathies, and neurons are specifically vulnerable to mitochondrial dysfunction, because they depend on efficient energy metabolism throughout the entire axon length [49, 50]. Other ubiquitously-expressed CMT2 genes involved in mitochondrial function include *MFN2*, *GDAP1 HSPB1* and *HSPB8* [50, 51]. Remarkably, the most downregulated protein in our analysis, COX6A1, situated in the mitochondrial complex IV, has previously been implicated in CMT. Recessive mutations in *COX6A1* cause slowly progressive axonal or mixed axonal and demyelinating neuropathy with childhood onset. *COX6A1* expression was significantly-reduced in peripheral blood cells from these patients [42].

AlaRS is not expected to impact mitochondrial translation directly, because it is a cytosolic protein [1, 2]. In contrast, a specialized AlaRS encoded by *AARS2* is expressed in mitochondria and is responsible for alanine incorporation into the mitochondrial proteins. Thus, we speculate that the observed mitochondrial involvement in monoallelic *AARS1* neuropathy could potentially be a secondary effect or alternatively, that altered synthesis of mitochondrial proteins in the cytosol could affect mitochondrial function in patients with *AARS1* neuropathy. Acquired mitochondrial dysfunction is a known mechanism underlying many adult-onset neurodegenerative diseases [52]. Whether this is the case also for CMT2 linked to *AARS1* p.Arg326Trp variant, as suggested by our data, should be explored in future studies.

IPA analysis in this study suggested that the acute phase response signalling pathway could play a significant role, and we speculated that this pathway is triggered by tissue injury in *AARS1* variant-positive patients. Maintenance and homeostasis in the nervous system, both in normal and disease states involves major input from the immune system [53]. It is also known that inflammation impact on mitochondrial biogenesis through reactive oxygen and nitrogen species, leading to oxidative stress and mitochondrial dysfunction [54]. In a recent study, Jennings et al., assessed biomarkers in the sera of 55 CMT patients, including six *AARS1* patients. They found an increase in NCAM1 and GDF15 and a widespread activation of the inflammatory complement system, of which especially C3 was upregulated among the CMT2 patients [55]. In our study, C3 was the top fourth upregulated protein and we saw an activation of proteins involved in inflammation. It was speculated in the study by Jennings et al., that neuromuscular junction degeneration may cause upregulation of the imflammatory system [55]. Unfortunately, neither NCAM1 or GDF15 were among the detected proteins in our proteomic screen based on PBMCs.

Further, the IPA graphical summary (Fig. 5B) showed that the transcription factor E2F1 is activated. Recently, it was shown in a *Drosophila* model of CMT that *YARS* mutants induced conformational changes in tyrosyl-ARS, leading to E2F1 hyper-activation [43]. With the caveat that the present analyses were performed in PBMC instead of neuronal tissue, where disease pathology manifest, and the sample number (*n* = 4) is very small, the data suggest that the *AARS1* mutation might also cause hyper activation of E2F1.

## Conclusion

This study provides a clinical and neurophysiological description of two large families who carry the p.(Arg326Trp) *AARS1* mutation. Proteomic data from four affected individuals suggest that mitochondrial dysfunction and inflammation could be involved in *AARS1* neuropathy. However, because these studies were performed in PMBC from four affected individuals, they should be confirmed by additional more highly-powered molecular, neurological and metabolic studies in nerve tissue.

## Supplementary Information

**Additional file 1.** Gene list. Peripheral neuropathy genes included in the analysis.

**Additional file 2.** Statistical analysis of the proteome of affected individuals compared to healthy controls. Sheet Perseus ALL shows the full comparison and sheet ‘STAT SIG’ the statistically significant differences between the *AARS1* patients and the controls. The sheet ‘STAT SIG’ has been sorted by column ‘difference’ showing how big the difference between the groups are (log10 values).

**Additional file 3.** Biochemical analysis of L-alanine. Biochemical analysis of L-alanine levels in total plasma for seven affected individuals (black squares to the left) versus nine controls (black circles to the right). The L-alanine levels are shown as percentage of average concentration in control samples.

**Additional file 4.** Canonical pathways in Ingenuity Pathway Analysis (IPA). The IPA analysis showed that 398 canonical pathways were significantly affected.

## Data Availability

The datasets used and analysed during the current study are available in the Additional files or from the corresponding author on reasonable request. The *AARS1* and *ATL1* variants have been submitted to the ClinVar database (https://www.ncbi.nlm.nih.gov/clinvar/), accession number SCV001571642 and SCV001571643 respectively. The mass spectrometry proteomics data have been deposited to the ProteomeXchange Consortium via the PRIDE partner repository (https://www.ebi.ac.uk/pride/) with the dataset identifier PXD023842 [56].

## Abbreviations

AARS1: Alanyl-tRNA synthetase
ARS: Aminoacyl-tRNA synthetase
CMAP: Compound motor action potential
CMT: Charcot-Marie-Tooth
CMT2: Charcot-Marie-Tooth type 2
dHMN: Distal hereditary motor neuronopathy
FC: Fold change
HGMD: The Human Gene Mutation Database
HMSN: Hereditary motor and sensory neuropathy
HSP: Hereditary spastic paraplegia
IPA: Ingenuity Pathway Analysis
MCV: Motor nerve conduction velocity
mRNA: Messenger RNA
NIS: Neuropathy impairment score
NGS: Next-Generation Sequencing
PBMC: Peripheral blood mononuclear cells
TCA: Tricarboxylic acid
VUS: Variant of unknown significance
