# Supplementary material for: Clinical characteristics and proteome modifications in two Charcot-Marie-Tooth families with the AARS1 Arg326Trp mutation
Source: BMC Neurol. 2022 Aug 15;22:299. doi: 10.1186/s12883-022-02828-6 (PMC9377087; doi:10.1186/s12883-022-02828-6)
Supplement: Supplementary file 1 — Additional file 1. Gene list. Peripheral neuropathy genes included in the analysis. [file 12883_2022_2828_MOESM1_ESM.docx]

**Additional file 1 – Gene lists**

Peripheral neuropathy genes included in the analysis.

During the bioinformatic filtering 52, 91 or 99 genes relevant to peripheral neuropathies were included and analysed. (The number of genes varied due to different time of analysis (2012, 2014 and 2016).

NGS gene panel with 52 neuropathy genes:

*AARS, ARHGEF10, ATL1, ATP7A, BSCL2, CTDP1, DCTN1, DNM2, DYNC1H1, EGR2, FAM134B, FGD4, FIG4, GAN, GARS, GDAP1, GJB1, HK1, HSPB1,HSPB3, HSPB8, IGHMBP2, IKBKAP, KIF1B, LITAF, LMNA, MED25, MFN2, MPZ, MTMR2, NDRG1, NEFL, NGF, NTRK1, PLEKHG5, PMP22, POLG, PRPS1, PRX, RAB7,REEP1, SBF2, SEPT9, SETX, SH3TC2, SLC12A6, SOD1, SOX10, SPTLC1, TRPV4, WNK1, YARS.*

Analysed for the proband in family 1 (II.2) and his sister (II.3).

NGS gene panel with 91 neuropathy genes:

*AARS, AIFM1, ARHGEF10, ATL1, ATL3, ATP7A, BICD2, BSCL2, CCT5, CTDP1, DCTN1, DCTN2, DHTKD1, DNAJB2, DNM2, DNMT1, DST, DYNC1H1, EGR2, FAM134B, FBLN5, FBXO38, FGD4, FIG4, GAN, GARS, GDAP1, GJB1, GJB3, GNB4, HARS, HINT1, HK1, HMCN1, HOXD10, HSPB1, HSPB3, HSPB8, IFRD1, IGHMBP2, IKBKAP, INF2, KARS, KIF1A, KIF1B, KIF5A, LITAF, LMNA, LRSAM1, MARS, MED25, MFN1, MFN2, MPZ, MTMR2, MYH14, NDRG1, NEFL, NGF, NTRK1, PDK3, PLA2G6, PLEKHG5, PMP22, POLG, PRNP, PRPS1, PRX, RAB7A, REEP1, SBF1, SBF2, SCN11A, SCN9A, SEPT9, SETX, SH3TC2, SLC12A6, SOD1, SOX10, SPTLC1, SPTLC2, SURF1, TFG, TRIM2, TRPV4, TUBB3, VAPB, VRK1, WNK1, YARS.*

Analysed for the proband in family 2 (II.1).

NGS gene panel with 99 neuropathy genes:

*AARS, AIFM1, ARHGEF10, ATL1, ATL3, ATP7A, BICD2, BSCL2, CCT5, COX6A1, CTDP1, DCAF8, DCTN1, DCTN2, DHTKD1, DNAJB2, DNM2, DNMT1, DST, DYNC1H1, EGR2, FAM134B, FBLN5, FBXO38, FGD4, FIG4, GAN, GARS, GDAP1, GJB1, GJB3, GNB4, HARS, HINT1, HK1, HOXD10, HSPB1, HSPB3, HSPB8, IGHMBP2, IKBKAP, INF2, KARS, KIF1A, KIF1B, KIF5A, LAMA2, LITAF, LMNA, LRSAM1, MARS, MED25, MFN2, MME, MORC2, MPZ, MTMR2, MYH14, NAGLU, NDRG1, NEFL, NGF, NTRK1, PDK3, PLA2G6, PLEKHG5, PMP22, POLG, PRNP, PRPS1, PRX, RAB7A, REEP1, SBF1, SBF2, SCN11A, SCN9A, SEPT9, SETX, SH3TC2, SLC12A6, SLC25A46, SLC5A7, SOD1, SOX10, SPG11, SPTLC1, SPTLC2, SURF1, TDP1, TFG, TRIM2, TRPV4, TUBB3, VAPB, VCP, VRK1, WNK1, YARS.*

Analysed for the proband in a third family who was later connected to family 2. Individual III.2 in family 2.
