# Supplementary material for: Clinical characteristics and proteome modifications in two Charcot-Marie-Tooth families with the AARS1 Arg326Trp mutation
Source: BMC Neurol. 2022 Aug 15;22:299. doi: 10.1186/s12883-022-02828-6 (PMC9377087; doi:10.1186/s12883-022-02828-6)

Additional file 3 - Biochemical analysis of L-alanine

Biochemical analysis of L-alanine levels in total plasma for seven affected individuals (black squares to the left) versus nine controls (black circles to the right). The L-alanine levels are shown as percentage of average concentration in control samples.

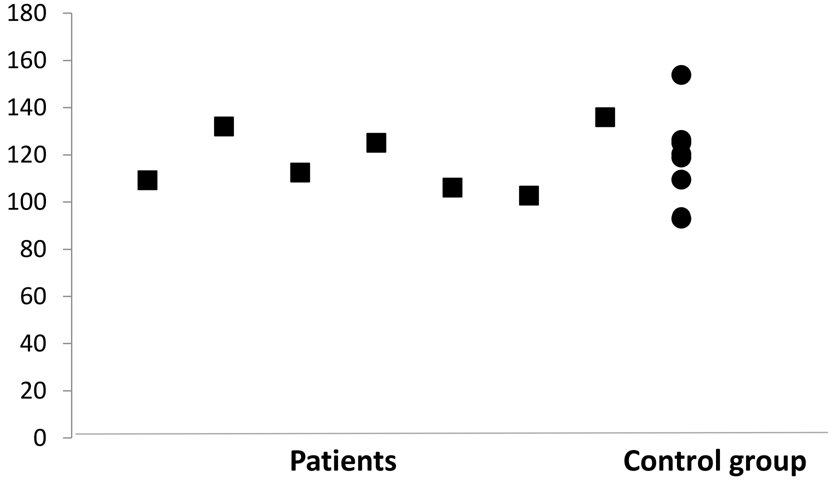

Supplement: Supplementary file 3 — Additional file 3. Biochemical analysis of L-alanine. Biochemical analysis of L-alanine levels in total plasma for seven affected individuals (black squares to the left) versus nine controls (black circles to the right). The L-alanine levels are shown as percentage of average concentration in control samples. [file 12883_2022_2828_MOESM3_ESM.pdf]
